# Iterative clustering algorithm G-DESC-E and pan-cancer key gene analysis based on single-cell sequencing data

**DOI:** 10.1093/bib/bbaf288

**Published:** 2025-07-03

**Authors:** Ke Wu, Changming Sun, Jie Geng, Ping Wang, Qi Dai, Leyi Wei, Ran Su

**Affiliations:** School of Computer Software, College of Intelligence and Computing, Tianjin University, Tianjin, China; CSIRO Data61, NSW 1710, Australia; Department of Cardiology, Chest Hospital, Tianjin University, Tianjin, China; Tianjin Key Laboratory of Cardiovascular Emergency and Critical Care, Tianjin Municipal Science and Technology Bureau, Tianjin, China; Tianjin Modern Innovative TCM Technology Co. Ltd, Tianjin 300392, China; College of Life Science and Medicine, Zhejiang Sci-Tech University, Hangzhou 310018, China; Centre for Artificial Intelligence driven Drug Discovery, Faculty of Applied Science, Macao Polytechnic University, Macao SAR, China; School of Computer Software, College of Intelligence and Computing, Tianjin University, Tianjin, China

**Keywords:** single-cell sequencing, deep learning, clustering, pan-cancer analysis

## Abstract

Single-cell sequencing technology has profoundly revolutionized the field of cancer genomics, enabling researchers to explore gene expression profiles at the resolution of individual cells. Despite its extensive applications in the study of cancer gene states, pan-cancer analyses remain relatively underexplored. In this study, we propose the G-DESC-E algorithm, which effectively distinguishes dimensionality-reduced data through a grid-based approach, filters out outliers during the preprocessing phase, and employs the Louvain algorithm for prescreening cluster centroids as initial clusters. We construct an objective function by integrating label entropy with the Kullback–Leibler divergence formula, achieving final clustering results through iterative optimization. Our findings demonstrate the effectiveness of the G-DESC-E algorithm in enhancing clustering accuracy. By applying our methodology to real-world datasets, we illustrate its capability to identify critical transcriptional features associated with distinct cancer subtypes. Coupled with clustering visualization and gene ontology analysis, we identify over thirty genes potentially related to cancer occurrence and progression. The algorithm and research framework presented in this study pave the way for new directions in clinical research by applying single-cell sequencing technology to the analysis of key genes within the realm of pan-cancer analysis for the first time. This approach offers valuable insights that can inform further clinical investigations.

## Introduction

Gene testing plays a pivotal role in early cancer screening and targeted therapy [1]. Understanding the alterations in gene expression levels and their effects in the human body, elucidating interactions between genes, and accurately identifying key genes that are strongly associated with cancer onset are essential for advancing cancer research. However, conducting in-depth studies of cancer cell marker genes using traditional gene sequencing methods poses significant challenges. The emergence of single-cell sequencing technology has facilitated more comprehensive investigations into cancer cell marker genes [2].

In the early stages of its development, single-cell sequencing data analysis was employed to investigate various cellular activities, including the construction of human cell landscapes and the study of cellular growth, as noted by Zhou *et al*. [3]. In 2020, Greenwald and colleagues harnessed single-cell sequencing technology to analyze pan-cancer datasets, which led to the identification of recurring patterns of cellular heterogeneity [4]. This pivotal work effectively integrated single-cell sequencing into the domain of pan-cancer research. Subsequently, the application of single-cell sequencing data in cancer studies became increasingly prevalent. In 2021, Zhou and collaborators elucidated the lineage differentiation of human lung adenocarcinoma cells using single-cell sequencing technology, thereby providing valuable insights for the clinical early diagnosis and stage-specific metastasis of lung adenocarcinoma [5]. Following this, various research teams undertook related investigations focused on cancer recurrence, prognostic modeling, and predictions of responses to cancer therapeutics [5–10]. However, as single-cell sequencing technology has matured and the volume of sequencing data has continued to expand, cluster analysis has encountered new challenges [11]. One significant challenge, closely associated with computational biology, is the enhancement of clustering accuracy.

The accuracy of clustering is influenced by the characteristics of the data and the selection and fine-tuning of clustering algorithms [12]. Conventional clustering methods, such as K-means and Louvain, often select outlier points as initial seeds, which are relatively distant from other data points. To mitigate this issue, Goldwasser *et al*. (2022) introduced a forest fire clustering method specifically designed for single-cell sequencing [13]. This innovative approach integrates iterative label propagation with parallelized Monte Carlo simulations. By employing an iterative cycling process and incorporating the concept of label entropy, the method computes non-parametric pointwise posterior exclusion probabilities for internal validation. This strategy effectively reduces the impact of outliers, thereby ensuring the algorithm’s stability and scalability; however, it also significantly increases resource consumption.

Another significant factor influencing clustering accuracy is the presence of batch effects, which are defined as variations in gene expression that occur between different measurement batches [14]. Traditionally, the processes of removing batch effects and performing clustering have been carried out as separate tasks via bioinformatics platforms such as Seurat and Scanpy. These platforms employ methodologies such as canonical correlation analysis and mutual nearest neighbors to reduce batch effects prior to implementing clustering algorithms such as Louvain for cluster analysis. However, the intricate interplay between clustering and batch effects removal may result in the retention of batch effects specific to certain cell types or conversely lead to the over-correction of these effects.

Conventional methods for the removal of batch effects include Harmony, Seurat 3, and LIGER(linked inference of genomic experimental relationships). These methodologies have been validated by Tran *et al*. to be highly effective in terms of overall performance and positioning batch effects removal within the data preprocessing phase [15]. In 2020, Lyu and colleagues introduced an unsupervised deep learning algorithm known as DESC, which utilizes the Kullback–Leibler (KL) divergence as its objective function to iteratively eliminate batch effects through deep learning techniques [16]. Their research findings provide a detailed explanation of the advantages of selecting KL divergence as the metric for batch effects elimination. This approach effectively removes complex batch effects while preserving biological variability, and empirical evidence suggests that it outperforms traditional methods for batch effects removal. However, it is important to note that this algorithm primarily targets the enhancement of clustering accuracy from the standpoint of batch effects removal, rendering it less effective for datasets devoid of batch effects or those characterized by weak batch effects.

Recognizing that batch effects are not the sole determinant of clustering accuracy, it becomes imperative to simultaneously enhance clustering accuracy and mitigate batch effects. To address this challenge, we propose a novel method termed G-DESC-E, which employs a grid-based preprocessing strategy [12] that partitions the clustering regions and establishes a density threshold to calculate the density of each point while effectively excluding outliers during the initial phase. Subsequently, by integrating the concept of label entropy [13] into the objective function of the DESC algorithm, we systematically improve clustering accuracy while concurrently reducing batch effects.

In comparative experiments, we confirmed its superiority in single-cell data analysis over other conventional clustering methods and deep learning clustering algorithms by utilizing the adjusted rand index (ARI) as a performance metric [17]. This study selected the widely used algorithms Leiden [18] from the Scanpy platform and the unmodified DESC [16] model as benchmarks for comparison. Experiments conducted on multiple datasets revealed that G-DESC-E exhibits superior scalability and clustering accuracy compared to DESC, while maintaining equivalent stability and resource consumption.

In the realm of downstream analysis, Maynard and others, along with Xie from Peking University Cancer Hospital, have investigated the origins and evolutionary processes of lung cancer and breast cancer cells, respectively, employing visualized clustering images [6, 7]. Gao and his colleagues utilized single-cell RNA sequencing technology to analyze malignant cell populations and developed prognostic models for lung adenocarcinoma [10]. However, the effects of various highly expressed genes and their relationship with cancer onset remain unclear.

In this study, we proposed the G-DESC-E algorithm. Initially, we employed a grid preprocessing method to eliminate outliers prior to formal clustering, thereby reducing resource consumption. Subsequently, we combined the KL divergence formula with label entropy to design an objective function, and utilized iterative optimization methods to progressively mitigate batch effects, enhancing clustering accuracy. In comparative experiments, our algorithm demonstrated impressive performance and effectiveness. We then conducted a thorough analysis of the clustering visualization results, integrating gene ontology (GO) [19] analysis to identify potential marker genes indicative of cellular malignancy and elucidate the relationships between biological processes. Additionally, through the analysis of gene distribution maps across multiple cancer and pan-cancer datasets, we explored the relationship between gene expression levels and cancer development. Based on single-cell sequencing data, this study presents over ten genes that may indicate cancer occurrence or variation within the pan-cancer context for the first time, and builds upon previous research by proposing more than ten key genes associated with four specific cancers, thereby providing valuable insights for clinical predictions and variations in cancer manifestation.

## Materials and methods

### G-DESC-E algorithm

#### Introduction to the DESC algorithm

The DESC algorithm is an unsupervised deep learning method that employs the KL divergence as its objective function. This algorithm systematically mitigates batch effects during the iterative process, thereby enhancing clustering accuracy. However, this improvement is contingent upon the technical variations between different batches in the dataset being less than the true biological differences, such as those observed between distinct cell types [16].

#### Parameter initialization in the G-DESC-E algorithm

The G-DESC-E algorithm commences its parameter initialization process by transforming the single-cell sequencing gene expression matrix, denoted as $M$ and dimensioned in $\mathbb{R}^{c\times g}$, where $c$ represents cells and $g$ denotes genes. This critical step involves transitioning from the high-dimensional gene space $\mathbb{R}^{g}$ to a significantly more condensed low-dimensional space $\mathbb{R}^{d}$, with the constraint that $d$ is substantially less than $g$. This transformation addresses the inherent sparsity and vast dimensionality of the gene expression data. A stack of autoencoders serves as the feature transformation mechanism, which has been empirically shown to enhance cluster separation in actual datasets over conventional dimensionality reduction techniques [17].

#### Stacked autoencoder architecture for feature transformation

**Figure 1:**
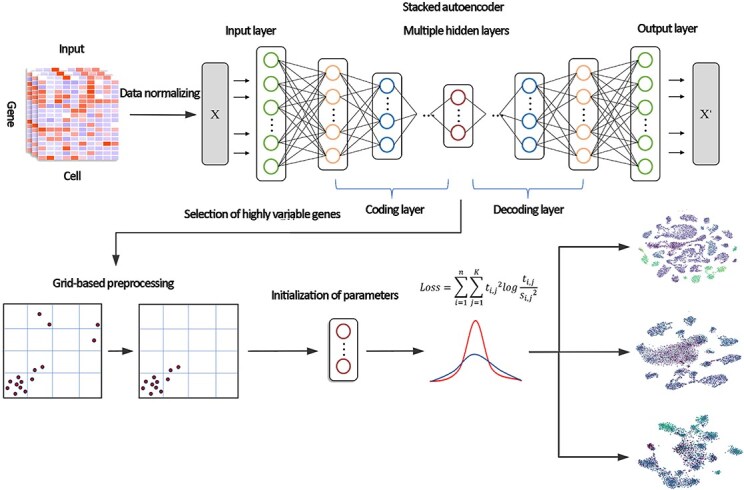
Overview of the our framework. Input the expression matrix of single-cell sequencing datasets and preprocess the data for dimensionality reduction, normalization, and selection of highly variable genes before performing clustering with an objective function optimization to generate a clustering image indicating gene expression levels.

In the context of the G-DESC-E algorithm, the stacked autoencoder [20] is meticulously crafted with layer-wise initialization aimed at reconstructing the output of its preceding layer. Each tier builds upon the previous one, forming a hierarchical structure that undergoes fine-tuning in reverse order to minimize reconstruction loss. The architecture, delineated in Fig. 1, consists of several layers culminating in an intermediate bottleneck layer. This pivotal layer serves as the reduced feature space, while the subsequent decoder layer endeavors to project back to the original high-dimensional data space.

#### Normalization of single-cell gene expression data

Prior to clustering, the G-DESC-E algorithm performs a critical normalization procedure on the gene expression data. Initially, each gene’s unique molecular identifier (UMI) count per cell is normalized by that cell’s total UMI count, followed by a scaling factor of 10 000; this facilitates conversion to a logarithmic scale at the individual cell level. Subsequently, gene-level normalization takes place, entailing the subtraction of the mean expression value across all cells and division by the standard deviation for each gene. In cases where batch effects are present within the dataset, the algorithm ensures that normalization is conducted within each specified cell batch to mitigate potential biases introduced during sample processing.

#### Identification of highly variable genes

To discern highly variable genes within single-cell sequencing data, researchers employ the “filter genes dispersion” function provided by the Scanpy package. This function is specifically designed to isolate genes that exhibit significant levels of expression variability across the cell population, which are pivotal in distinguishing cellular heterogeneity.

#### Grid-based data preprocessing

Following dimensionality reduction, the preprocessing step necessitates the segmentation of data points into a lattice of grids. Designating $p$ as the total number of grids and utilizing $d_{t}$ as a density threshold, a grid is considered dense if data points $G(i)$ contained within it surpass threshold $d_{t}$. For grids with $0 < G(i) \leq d_{t}$, those data points that maintain a Euclidean distance greater than half the diagonal length from their nearest dense counterpart are flagged as outliers. The formula for the Euclidean distance is as follows:


\begin{align*}& D = \sqrt{(x_{a} - x_{b})^{2} + (y_{a} - y_{b})^{2}}. \end{align*}

Where $x_{a}$ and $x_{b}$ are the $x$-coordinates of the two-dimensional data points after dimensionality reduction, $y_{a}$ and $y_{b}$ are the $y$-coordinates of the data points, and $D$ is the Euclidean distance between the two data points. The iterative refinement process continues by purging isolated points until no further outliers are detected.

The selection of grid size is primarily based on the sparsity of data distribution, the objectives of downstream analysis, and computational efficiency. When data points are sparsely distributed and the analytical goal is to identify global patterns, a relatively larger grid size should be chosen; conversely, the choice of grid size should accommodate a smaller size if the goal is to capture more localized structures. Additionally, the balance between computational efficiency and accuracy must be considered in the selection process. The setting of the density threshold determines the number of identified outliers, which is crucial for ensuring the model’s effectiveness. A higher threshold may lead to the omission of potential clusters, while a lower threshold can capture more local structures but may also introduce noise. Consequently, the selection of grid size and threshold can be continually adjusted through methods such as cross-validation in experiments to obtain optimal parameters that align with the research objectives.

#### Cluster center selection for single-cell data

Given the absence of definitive cluster information in single-cell sequencing datasets, initial selection of cluster centroids is essential. The algorithm adopts the Louvain method to derive feature space $F$ from the autoencoder’s bottleneck layer, thus determining the cluster quantity $K$ along with the initial cluster centroids ($k_{j}, j = 1, \dots , K$). This graph-based clustering technique has been recognized for its superiority over other methods.

#### The process of iterative clustering

Upon completion of preprocessing and centroid determination, the dataset undergoes a series of alternating processing steps aimed at achieving convergence. Initially, the similarity between each cell $i$’s embedding point $f_{i}$ and the centroid $k_{j}$ of cluster $j$ is gauged using the Student’s t-distribution [13] as a kernel:


(1) \begin{align*}& s_{i,j}=\frac{\left(1+\frac{\|f_{i}-k_{j}\|^{2}}{\alpha}\right)^{-1}}{\sum_{j}\left(1+\frac{\|f_{i}-k_{j}\|^{2}}{\alpha}\right)^{-1}}.\end{align*}

Here, $f_{i} = f_{W(m_{i})}$ is situated within the feature space $F$, with $m_{i}$ originating from matrix $M$ post-embedding. The parameter $\alpha $ signifies the degree of freedom within the Student’s t-distribution. Subsequently, the target function amalgamates the KL divergence loss and label entropy as per Equation (2), striving to optimize clustering outcomes via the minimization of the encoding layer’s loss:


(2) \begin{align*}& Loss=\sum_{i=1}^{n}\sum_{j=1}^{K}t_{i,j}^{2}\log\frac{t_{i,j}}{s_{i,j}^{2}}.\end{align*}

The cell $i$’s soft clustering distribution is denoted as $s_{i,j}$, $t_{i,j}$ represents the auxiliary distribution, and $n$ standing for the cell count. The auxiliary distribution $t$ is defined as follows:


(3) \begin{align*}& t_{i,j}=\frac{\frac{s_{i,j}^{2}}{\sum_{i=1}^{n}s_{i,j}}}{\sum_{j=1}^{K}\left(\frac{s_{i,j}^{2}}{\sum_{i=1}^{n}s_{i,j}}\right)}.\end{align*}

With $\alpha $ typically set to 1, the auxiliary distribution $t$’s role is to enhance the purity of clusters by prioritizing cells with high confidence levels. The $t_{i,j}$ value indicates the probability of cell $i$ belonging to cluster $j$, serving as a measure of the confidence level associated with that cell’s cluster assignment.

#### Objective function optimization via stochastic gradient descent

In our experiment, the optimization process of the objective function is realized through stochastic gradient descent [21]. We concentrate on the gradients generated from the feature space embeddings of data points $f_{i}$ and cluster centroids $k_{j}$.


(4) \begin{align*} & {\partial Loss\over \partial f_{i}}={\alpha+1\over \alpha}\sum_{j=1}^{K}\left(1+\frac{f_{i}-k_{j}^{2}}{\alpha}\right)^{-1}\times (t_{i,j}-s_{i,j})(f_{i}-k_{j}). \end{align*}

(5) \begin{align*} &\ \ \ {\partial Loss\over \partial k_{j}}=-{\alpha+1\over \alpha}\sum_{i=1}^{n}\left(1+\frac{f_{i}-k_{j}^{2}}{\alpha}\right)^{-1}\times (t_{i,j}-s_{i,j})(f_{i}-k_{j}). \end{align*}

The computation of these gradients contributes to determining parameter gradients within the deep neural network during the well-established backpropagation technique. We execute model training under the Keras framework [21]. During this iterative procedure, updates to the auxiliary distribution $T$ are made unless losses descend below a pre-set epoch tolerance value. If such convergence criteria are met, iteration ceases. The standard tolerance value is configured at 0.005, calculated as per the formula:


(6) \begin{align*}& tol=\frac{\#|X_{curr}\neq X_{prev}|}{n}.\end{align*}

In this equation, $X_{curr}$ represents the cluster number defined by the highest probability of cluster assignment in the current iteration, while $X_{prev}$ refers to the cluster number from the previous iteration. Here, $\#|X_{curr} \neq X_{prev}|$ indicates the changing number of cells between consecutive iterations.

#### Deep neural network architecture

For the design of the neural network architecture in this study, we adapt the number of hidden layers and nodes to accommodate the dataset’s size. Employing a greater number of hidden layers and nodes may enable the neural network to more accurately represent the complexity inherent within the data. However, this increase also demands higher computational resources. In our configuration, the tanh activation function is applied to both bottleneck and decoder layers to ensure that the output values are normalized within a suitable range, while the ReLU activation is chosen for other structures to maintain non-linear properties essential for deep learning tasks [10, 22].

### Batch effects evaluation metric

Our approach utilizes $KL$ divergence to evaluate the effectiveness of algorithms in reducing batch effects. For datasets consisting of multiple batches, designated as $B$, the $KL$ divergence metric is computed as follows:


(7) \begin{align*}& KL=\sum_{b=1}^{B}p_{b}\log{p_{b}\over q_{b}}.\end{align*}

Within this calculation, $p_{b}$ signifies the proportion of cells in batch $b$ relative to the overall cell population, while $q_{b}$ illustrates the proportional cell distribution within a specific biological region, as determined by the outcome of the clustering algorithm. It holds true that:


(8) \begin{align*}& \left \{ \begin{aligned} \sum_{b=1}^{B}q_{b}=1,\\ \sum_{b=1}^{B}p_{b}=1. \end{aligned} \right .\end{align*}

A lower $KL$ divergence value is desirable, indicating a more successful mitigation of batch effects.

### Label entropy for clustering result optimization

In our methodological framework, label entropy is an instrumental metric to improve the clustering results derived from the algorithm. We calculate the label entropy for each cell $i$ using the formula:


(9) \begin{align*}& E_{i}=-\sum_{j=1}^{K}P_{i,j}\log(P_{i,j}),\end{align*}

where $P_{i,j}$ in this context is the probability of assigning cell $i$ to cluster $j$, mirroring the logic of Equation (3). Elevated levels of label entropy reflect a higher degree of uncertainty in the cluster assignments, suggesting a growing discrepancy from the ground truth [13].

The expression for label entropy $E$ relies solely on the probabilities $P_{i,j}$. By integrating this measure with the $KL$ divergence metric and comparing it to DESC’s initial objective function, the relevance of the weight factor $t_{i,j}$ as depicted in Equation (3) becomes apparent and necessitates amplification. Through extensive testing, we have fine-tuned the weighting enhancement ratio and solidified the objective function as demonstrated in Equation (2).

### Clustering evaluation metric: adjusted rand index

During our analysis, the ARI serves as the primary metric for evaluating the clustering performance of algorithms, given the availability of reference label assignments in the selected datasets. The ARI effectively measures the concordance of the inferred classification labels against the authentic labels. Specifically, this metric compiles the deduced cluster labels and known labels into a contingency table, enabling the examination of commonalities between both label sets. The ARI is succinctly expressed through the formula:


\begin{align*} &\textrm{ARI}= \frac{\sum_{u,v}({n_{uv}/ 2}) -\frac{[\sum_{u}({a_{u}}/{2})\sum_{v}({b_{v}}/{2})]}{({n_{uv}/ 2})} } {{1\over 2}[\sum_{u}({a_{u}/ 2})+\sum_{v}({b_{v}/ 2})]-\frac{[\sum_{u}({a_{u}}/{2})\sum_{v}({b_{v}}/{2})]}{({n_{uv}/ 2})} }. \nonumber\end{align*}

Here, $n_{uv}$ counts the cells jointly classified under cluster $u$ according to the true labels and cluster $v$ following the algorithmic labels. Meanwhile, $a_{u}$ and $b_{v}$ represent the sum of cells categorized under clusters $u$ and $v$, respectively, based on true labels and assigned cluster labels.

## Results

### Method overview

As illustrated in Fig. 1, the G-DESC-E algorithm initially identifies and removes isolated points from the dataset. The filtered dataset is then initialized using the parameters obtained from an autoencoder trained via deep neural networks. The algorithm integrates steps that are traditionally considered preprocessing stages in biological research, such as batch effects removal, into its iterative optimization process. The ultimate aim of this approach is to enhance the accuracy of clustering analysis.

We utilized the feature matrix of single-cell sequencing data, organized with cells as rows and genes as columns, as the input. After normalization and dimensionality reduction, we projected the data into points, which were then divided into grids. We established a corresponding number of grids and density thresholds to differentiate between dense data points and isolated ones. Through multiple iterations aimed at removing isolated points, our objective is to reduce uncertainties and enhance the resource efficiency of subsequent clustering tasks.

After preprocessing, the G-DESC-E algorithm balanced the biological and technical differences among different clusters during the iterative clustering process by relocating cells to the nearest cluster centroids, thereby mitigating the batch effects. Furthermore, to further improve clustering accuracy, we have designed an objective function that integrates KL divergence and information entropy. The resulting clustering outcomes provide valuable insights for exploring the roles of relevant biological processes and key genes in cancer.

### Evaluation of G-DESC-E clustering based on multiple datasets

**Figure 2:**
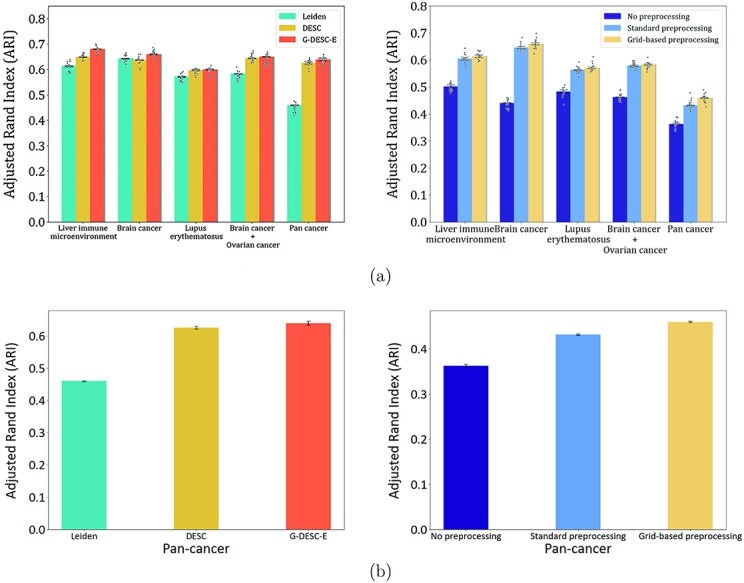
Comparison between different methods. (a) The left panel displays the ARI values for four distinct datasets (liver immune microenvironment, brain cancer, lupus erythematosus, and a combination of brain cancer and ovarian cancer) clustered using Leiden, DESC, and G-DESC-E methods. The right panel presents the ARI values for these same datasets clustered using no preprocessing, standard preprocessing, and grid-based preprocessing. The calculation of p-values presented here utilizes the one-way ANOVA method, which will be consistently applied throughout the following sections. (b) The left panel displays the ARI values for the pan-cancer dataset clustered using Leiden, DESC, and G-DESC-E methods. The right panel shows the ARI values for the pan-cancer dataset clustered using no preprocessing, standard preprocessing, and grid-based preprocessing. The pan-cancer dataset includes five types of cancers: brain cancer, breast cancer, lung cancer, ovarian cancer, and colorectal cancer. The error bars were calculated based on six replicate experiments.

In order to evaluate the performance of the G-DESC-E algorithm, we conducted clustering analysis on seven single-cell sequencing datasets. These datasets included liver immune microenvironment data [23], a lupus erythematosus dataset [24], and five cancer datasets derived from the 10x Genomics platform [25]. We compared our G-DESC-E with two commonly used methods, Leiden [18] and DESC [16]. The Leiden algorithm is an enhanced version of the Louvain algorithm, designed to perform community detection in graph structures by optimizing modularity. This approach effectively avoids local optima and improves the quality of community partitioning. In contrast, the DESC algorithm is a deep learning-based method leveraging KL divergence, employing iterative optimization to repeatedly refine the clustering process while mitigating the batch effects. The Leiden algorithm focuses on optimizing modularity within graph structures, whereas DESC emphasizes a density-driven clustering approach. The G-DESC-E algorithm builds upon DESC, further refining the objective function to reduce batch effects while enhancing clustering accuracy. Additionally, we conducted a separate evaluation of the performance of the grid-based preprocessing method. We compared its results with those data that underwent no preprocessing and data processed using traditional preprocessing methods. The traditional preprocessing steps included filtering out cells with a gene count below a certain threshold, removing genes with expression levels below a specified threshold, normalizing the data, selecting highly variable genes, applying the ComBat algorithm [14] to eliminate batch effects, and performing dimensionality reduction. For all three types of data, we utilized the Leiden algorithm for clustering and employed the ARI as the evaluation metric, as illustrated in Fig. 2, with specific values shown in Table 1.

**Table 1:** Comparison between different methods

Category	No preprocessing	Standard preprocessing	Grid-based preprocessing
Liver immune microenvironment	0.50219(0.00677)	0.60481(0.00535)	0.61337(0.00466)
Brain cancer	0.44062(0.00259)	0.64553(0.00413)	0.65971(0.00407)
Lupus erythematosus	0.48372(0.00579)	0.56308(0.00193)	0.57144(0.00327)
Brain cancer + Ovarian cancer	0.46238(0.00141)	0.57833(0.00295)	0.58324(0.00187)
Pan-cancer	0.36256(0.00361)	0.43210(0.00187)	0.46009(0.00154)
**Category**	**Leiden**	**DESC**	**G-DESC-E**
Liver immune microenvironment	0.61337(0.00183)	0.64873(0.00123)	0.68062(0.00113)
Brain cancer	0.64327(0.00167)	0.63698(0.00122)	0.65971(0.00124)
Lupus erythematosus	0.57144(0.00186)	0.59774(0.00216)	0.60023(0.00221)
Brain cancer + Ovarian cancer	0.58324(0.00052)	0.64417(0.00153)	0.64988(0.00121)
Pan-cancer	0.46009(0.00122)	0.62507(0.00045)	0.63941(0.0058)

The upper part of the table presents the ARI obtained by clustering five datasets using no preprocessing, standard preprocessing, and grid-based preprocessing. The lower part of the table displays the ARI for the five datasets clustered using Leiden, DESC, and G-DESC-E methods. The numbers in parentheses represent the *P*-values corresponding to the respective methods and datasets.

The results clearly indicate that data preprocessing plays a crucial role in enhancing clustering accuracy. The grid-based preprocessing method demonstrated effectiveness in improving the ARI values of the experimental datasets compared to traditional preprocessing algorithms, regardless of the presence of significant batch effects. However, in datasets without notable batch effects (such as the brain cancer dataset), the clustering accuracy of the DESC algorithm was inferior to that of traditional methods. This discrepancy arises because the DESC algorithm primarily focuses on mitigating batch effects while neglecting other factors relevant to clustering. In contrast, the G-DESC-E algorithm outperformed both the Leiden and DESC algorithms in terms of clustering accuracy, particularly on datasets exhibiting pronounced batch effects, such as those from the pan-cancer cohort.

### Pan-cancer key gene analysis

In order to investigate the role of gene expression levels in cancer prediction and provide clinical references, we employed the G-DESC-E algorithm to cluster a pan-cancer dataset and selected 15 genes from the visualization of the top 3000 expressed genes that were significantly expressed across all clusters in the pan-cancer dataset, conducting an in-depth analysis of these genes. The gene selection method for subsequent analyses focusing on individual cancer types closely mirrors this approach. During the identification of effective cancer predictive genes, We initially excluded ATPAF1 (associated with mitochondrial function) and ECHDC1 (involved in fatty acid oxidation) from our analysis because the expression levels of these functional genes are influenced by multiple factors. For instance, the expression level of ATPAF1 may be affected by oxygen levels and dietary habits, while the expression level of ECHDC1 can be influenced by exercise and dietary factors [26]. These factors may confound their direct relationship with cancer, rendering them unsuitable as cancer marker genes. Among the remaining 13 genes, chromodomain helicase DNA binding protein 6 (CHD6), FLII actin remodeling protein (FLII), tumor protein p53 inducible nuclear protein 2 (TP53INP2), and ral guanine nucleotide dissociation stimulator like 2 (RGL2) have been confirmed in recent studies to have elevated expression levels closely linked to oncogenesis; thus, we focused our analysis on the remaining nine genes: lysine demethylase 5C(KDM5C), CDC42 binding protein kinase beta (CDC42BPB), cytoskeleton-associated protein 4 (CKAP4), lysine methyltransferase 2D (KMT2D), PBX homeobox 2 (PBX2), retroelement silencing factor 1 (RESF1), family with sequence similarity 120A (FAM120A), BTB domain containing 9 (BTBD9), and ring finger protein 103 (RNF103).

**Figure 3:**
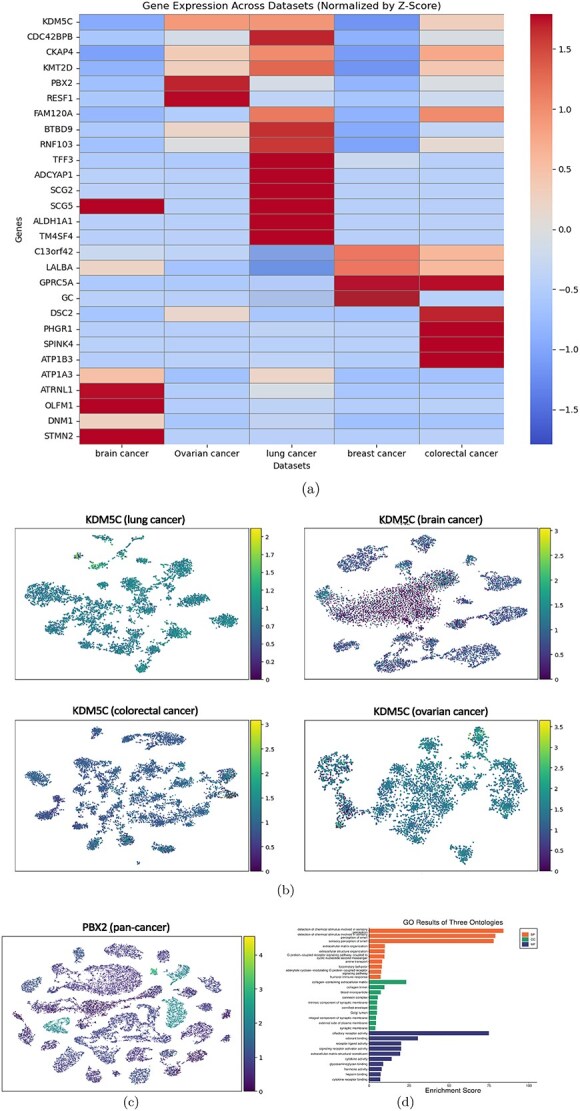
Multifaceted gene expression profiling across various cancer types. (a) This figure illustrates a comparison of the expression levels of various genes. (b) The figure depicts the expression status of KDM5C in lung cancer, brain cancer, colorectal cancer, and ovarian cancer, arranged from top left to bottom right. (c) The figure depicts the expression of PBX2 in a pan-cancer dataset. (d) The figure depicts the GO analysis generated from 3000 highly expressed genes within the pan-cancer dataset.

Based on the aforementioned nine potential pan-cancer marker genes and the individual cancer marker genes to be analyzed subsequently, we present the corresponding heatmap (Fig. 3a). This figure clearly illustrates the variations in expression levels of each gene across different cancers.

KDM5C is believed to promote cell growth by activating estrogen receptor target genes and evade immune surveillance by inhibiting type I interferons and interferon-stimulated genes, thereby facilitating the proliferation of breast cancer cells [26]. Additionally, increased expression of KDM5C has also been observed in four other cancer types (lung cancer, brain cancer, colorectal cancer, and ovarian cancer) (Fig. 3b), indicating its potential involvement in the progression of various cancer types and highlighting KDM5C as a key gene for cancer prediction beyond breast cancer.

Furthermore, CDC42BPB, CKAP4, KMT2D, and PBX2 have been shown to be strongly associated with the development of certain cancers [26]. Notably, all four members of the PBX family are linked to cancer. Figure 3c illustrates that PBX2 exhibits significantly high expression levels across the five analyzed cancers, suggesting its potential role as a biomarker for cancer prediction.

RESF1 is widely regarded as a crucial player in bacterial activities and the replication of viral RNA [26]. Several cancer-inducing factors stem from viral infections, while unavoidable complications during cancer treatment, such as tumor metastasis, organ compression, or chemotherapy, may lead to the overexpression of RESF1. Although utilizing RESF1 as a pivotal gene for cancer prediction could result in misunderstandings due to its ambiguous roles in various genetic processes, it is essential to further investigate its functions beyond cancer prediction, as this inquiry holds significant academic value.

The genes FAM120A, BTBD9, and RNF103 have not been previously linked to cancer [26]; rather, they have been associated with schizophrenia, restless leg syndrome, and depression, respectively. Notably, RNF103 serves as a molecular target for electroconvulsive therapy and antidepressant medications. Imaging studies suggest a potential relationship between these genes and cancer pathology.

Furthermore, to achieve a more intuitive understanding of gene programs that may be related to cancer, we selected the top 3000 highly expressed genes and generated GO images analyzed from three perspectives: molecular function (MF), cellular component, and biological process (BP), as illustrated in Fig. 3d.

GO enrichment analysis revealed that several BPs and MFs significantly affected in the dataset are closely associated with sensory perception, particularly olfaction. Among the enriched BPs, terms such as “detection of chemical stimulus involved in sensory perception,” “detection of chemical stimulus involved in sensory perception of smell,” and “sensory perception of smell” were highly represented. These processes suggest alterations in chemosensory pathways, which may reflect broader disruptions in physiological signaling caused by cancer progression. On the molecular function level, significant enrichment was observed in “olfactory receptor activity” and “odorant binding,” both of which are essential for the initial detection and transduction of odorant signals. The prominent enrichment of these olfaction-related GO terms suggests that tumor development may exert systemic effects extending beyond the primary site of malignancy, potentially impairing sensory systems. These findings prompt further investigation into whether cancer-induced changes in olfactory function could serve as early biomarkers or indicators of disease state.

### Key genes analysis in lung cancer

The pan-cancer dataset not only illustrates key genes across various cancer types but also identifies certain genes that are uniquely and highly expressed in specific cancers, potentially serving as indicators for particular malignancies. Here, we concentrated our analysis on several genes with notably high expression in lung cancer.

**Figure 4:**
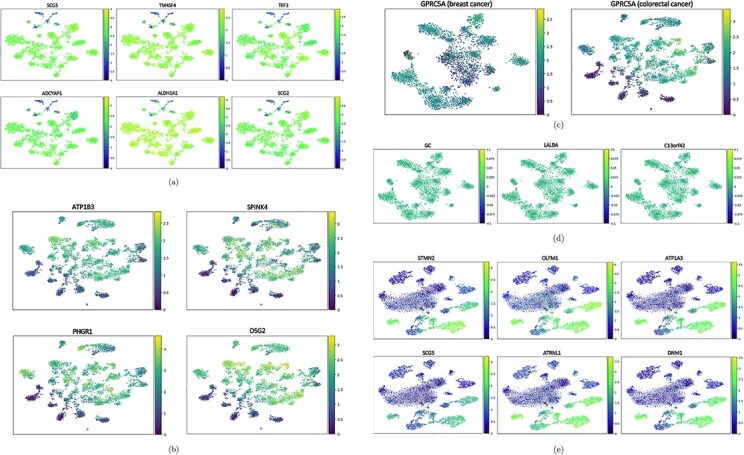
Cluster map of key cancer genes. (a) Display the expression levels of SCG5, TM4SF4, TFF3, ADCYAP1, ALDH1A1, and SCG2 within the lung cancer dataset. (b) Present the expression levels of ATP1B3, SPINK4, PHGR1, and DSG2 in the colorectal cancer dataset. (c) The plot displays the elevated expression levels observed in both breast cancer and colorectal cancer datasets. (d) The plot displays the expressions of GC, LALBA, and C13orf42 specifically in the breast cancer dataset. (e) The plot displays the distributions of STMN2, OLFM1, ATP1A3, SCG5, ATRNL1, and DNM1 in the brain cancer dataset.

Trefoil factor 3 (TFF3), a stable secretory protein, is associated with mucus production as well as mucosal stability and repair. Adenylate cyclase activating polypeptide 1 (ADCYAP1) plays a role in neuroendocrine stress responses by inducing an increase in intracellular calcium concentration. Secretogranin II (SCG2) has been confirmed to be related to primitive neuroectodermal tumors of the brain, while secretogranin V (SCG5) primarily regulates the secretion of pituitary hormones, preventing the aggregation of secretion proteins linked to certain diseases. Aldehyde dehydrogenase 1 family member A1 (ALDH1A1) is involved in metabolic regulation and is known to be associated with alcohol dependence and androgen insensitivity syndrome. Moreover, transmembrane 4 L six family member 4 (TM4SF4) contributes to cell cycle regulation, promoting cell proliferation. In prior research, these genes have not yet been validated as directly associated with lung cancer [26]. The SCG family has previously been implicated in various neurological disorders; however, experimental evidence indicates that their expression levels are significantly elevated in lung cancer datasets (Fig. 4a). Further control variables and comparative validations are required to ascertain whether these genes can serve as reliable indicators for the occurrence of lung cancer. Nonetheless, it is certain that they are closely linked to the onset and progression of this disease.

### Key genes analysis in breast and colorectal cancer

Chromosome 13 open reading frame 42 (C13orf42), lactalbumin alpha (LALBA), G protein-coupled receptor class C group 5 member A (GPRC5A), and GC vitamin D binding protein (GC) have been identified as the most highly expressed genes in breast cancer [26]. Previous studies have only associated GPRC5A with the early stages of lung carcinogenesis; however, our experiments indicate that GPRC5A is significantly overexpressed in both breast and colorectal cancers (see Fig. 4b). To date, C13orf42 has only been demonstrated to be related to Bardet-Biedl syndrome. As a monomer, LALBA exhibits a strong affinity for calcium and zinc ions, which may confer anti-tumor properties. Its folded variant has the potential to induce tumor cell lysis. The elevated expression of LALBA may suggest that breast cancer patients are actively undergoing treatment, thereby improving their clinical condition. Proteins encoded by the GC gene family are classified as transmembrane proteins, primarily responsible for transport functions. These proteins have previously been linked to the Wolf-Hirschhorn syndrome, a congenital chromosomal deletion disorder, and no direct connection to cancer development has yet been proposed (see Fig. 4c) [26].

While elevated expression levels of desmocollin 2 (DSC2) are generally associated with cardiovascular diseases [26], as illustrated in Fig. 4d, this gene is also significantly overexpressed in colorectal cancer. Diverticulitis of the colon is primarily linked to proline, histidine and glycine rich 1 (PHGR1), which may present either independently or as a complication of colorectal cancer [26]. Therefore, high expression levels of PHGR1 should not be interpreted as a definitive marker for colorectal cancer, but they may serve as supplementary reference indicators. Previous studies have suggested an association between serine peptidase inhibitor Kazal type 4 (SPINK4) and various subtypes of pancreatic cancer. The primary role of ATPase Na+/K+ transporting subunit beta 3 (ATP1B3) is to establish and maintain electrochemical gradients of sodium and potassium ions across the plasma membrane [26]. Related conditions include meningioma, with affected pathways encompassing cardiac conduction and infectious diseases. The relationships between these two genes and colorectal cancer merit further investigation. Additionally, it is noteworthy that several genes highly expressed in colorectal cancer are implicated in cardiac function. If this hypothesis is substantiated, it could provide valuable insights for predicting, treating, and prognosticating both cardiovascular diseases and colorectal cancer.

### Key genes analysis in brain cancer

The analysis of breast and colorectal cancers previously indicated that ATPase Na+/K+ transporting subunit alpha 3 (ATP1A3) is associated with meningiomas, and this gene also exhibits high expression levels in brain cancer datasets. Attractin like 1 (ATRNL1) is widely acknowledged for its role in the process of cell migration, with associated disorders including spinocerebellar ataxia. Currently, diseases related to olfactomedin 1 (OLFM1) include myopathy and neuroblastoma, with this gene showing significant expression in brain cancer datasets. Variants of dynamin 1 (DNM1) are typically linked to synaptic disorders, which may lead to developmental and epileptic encephalopathies. stathmin 2(STMN2) encodes a protein that regulates neuronal growth; notably, decreased expression of this gene is often correlated with Alzheimer’s disease and Down syndrome [26]. The SCG family has been established as relevant to brain diseases as highlighted in the analysis of key genes associated with lung cancer, and SCG5 was reconfirmed in the brain cancer dataset (see Fig. 4e).

Key genes that are significantly upregulated in brain cancer have been associated with various neurological disorders, indicating that their elevated expression may serve as predictive markers for the onset of these conditions. However, the symptoms associated with ATRNL1 and spinocerebellar ataxia are classified as chromosomal genetic disorders, which theoretically do not have a substantial relationship with brain cancer [26]. Therefore, the aberrant overexpression of certain genes may provide potential indicators for the diagnosis or progression of brain cancer in the future. In contrast, the role of genes such as OLFM1, which are also highly expressed in normal brain tissue, is less clear [26]. Moreover, aside from the observed increase in expression levels, atypical downregulation of some genes may also hold prognostic implications concerning cancer staging. These hypotheses necessitate further validation through comparative experimental studies in future research.

## Discussion

This study proposes a grid-based preprocessing technique aimed at reducing resource consumption and enhancing clustering accuracy. Additionally, the proposed method further improves clustering accuracy by optimizing the objective function of the DESC algorithm, thereby mitigating batch effects. Through GO analysis, this research identifies key genes associated with pan-cancer and specific cancer types, while also characterizing gene programs linked to these cancers.

The implementation of the grid-based preprocessing method in this study effectively eliminates certain outliers, which conserves resources during the clustering process, an aspect i.e. crucial for large-scale bioinformatics analyses. By systematically organizing data into grids, computational efficiency is significantly enhanced, resulting in improved clustering accuracy. This approach not only simplifies the computational workflow but also increases clustering fidelity, thereby underscoring the importance of preprocessing strategies in bioinformatics applications.

Moreover, the G-DESC-E algorithm represents a significant advancement in clustering methodologies. By refining the objective functions of the algorithm, this study enhances clustering accuracy while simultaneously addressing its limitations, particularly the diminished effectiveness observed when applied to datasets lacking batch effects. This improvement enables the G-DESC-E algorithm to achieve higher clustering accuracy than previous algorithms, thereby establishing its importance within the field of bioinformatics, where accurate clustering is crucial for deriving meaningful biological insights.

On the other hand, the identification of key genes associated with cancer mechanisms constitutes a major milestone in this research. The characterization of these critical genetic components provides valuable insights into the molecular mechanisms underlying cancer initiation and progression. A deeper understanding of the functions of these genes may facilitate breakthroughs in cancer diagnosis, prognosis, and potential therapeutic interventions, thereby making substantial contributions to the fields of oncology and precision medicine.

GO analysis on cancer-related genes provides a valuable perspective for understanding their functional roles and interactions within biological processes. By elucidating gene programs associated with cancer pathophysiology, this study offers a comprehensive view of the molecular events that drive tumorigenesis and disease progression. Such insights are crucial for unraveling the complexities of cancer biology and for identifying potential targets for future research and clinical applications.

In this study, the integration of grid-based preprocessing techniques, the design of algorithm objective functions, the identification of key cancer-related genes, and downstream analyses collectively deepen our understanding of cancer biology while enabling researchers to explore innovative avenues in cancer research. Ultimately, this work aims to provide improved diagnostic tools, targeted therapies, and personalized treatment strategies for clinical patients.

**Key Points**

- G-DESC-E is proposed a grid-density thresholding method to eliminate outliers in single-cell sequencing data prior to clustering. This approach reduces computational resource consumption while improving clustering accuracy (validated by ARI), addressing challenges posed by high-dimensional sparse data and noise.
- Developed a novel neural network model Grid-based Deep Embedding with Entropy for Single-cell Clustering (G-DESC-E) that unifies batch effects correction and clustering through iterative optimization. By incorporating label entropy and KL divergence into the objective function, the algorithm achieves superior performance in both batch effects mitigation (e.g. avoiding over-correction) and cluster resolution compared to traditional methods (e.g. DESC, Leiden) across multiple datasets.
- Identified >10 novel pan-cancer candidate genes and cancer-specific biomarkers (e.g. in lung adenocarcinoma and breast cancer) through integrated visualization and GO enrichment analysis. These genes exhibit expression patterns strongly correlated with tumorigenesis, providing mechanistic insights into cancer progression and potential targets for early diagnosis and therapy.
- Established a framework linking single-cell clustering outcomes to clinical interpretability. By mapping gene expression dynamics to altered biological processes (e.g. cell cycle regulation, and immune evasion), this work enhances understanding of cancer heterogeneity and informs personalized treatment strategies. Innovative grid-based preprocessing strategy for enhanced clustering efficiency.

## Data Availability

This study utilized a total of 7 publicly available datasets that can be accessed through the following accessions or URLs: (i) Lupus erythematosus dataset (GSE96583); (ii) Liver immune microenvironment dataset (GSE115469); (iii) Colorectal cancer data: Human Colorectal Cancer, https://www.10xgenomics.com/datasets/human-colorectal-cancer-11-mm-capture-area-ffpe-2-standard; (iv) Ovarian cancer data: Human Ovarian Cancer, https://www.10xgenomics.com/datasets/human-ovarian-cancer-11-mm-capture-area-ffpe-2-standard; (v) Breast cancer: https://www.10xgenomics.com/data sets/human-breast-cancer-visium-fresh-frozen-whole-transcriptome-1-standard; (vi) Lung cancer: https://www.10xgenomics.com/datasets/ human-lung-cancer-11-mm-capture-area-ffpe-2-standard and; (vii) Brain cancer: https://www.10xgenomics.com/datasets/human-brain-cancer-11-mm-capture-area-ffpe-2-standard. The lupus erythematosus dataset was generated by Targ *et al*. [23], obtained in general from SLE patients and two controls for HiSeq2500 data sequencing of PBMCs, totaling 14 619 entries used for algorithm performance evaluation. The liver immune microenvironment dataset was provided by Macparland *et al*. [24], derived from primary liver samples of 5 patients processed through 10$\times $ genomics sequencing, resulting in 8444 entries used for algorithm performance evaluation [25]. The brain, lung, breast, colorectal, and ovarian cancer datasets were sourced from the 10x genomics website, comprising 10 878, 6195, 4898, 9080, and 4674 cancer data entries respectively. Each dataset’s entries stem from the same batch of testing and are utilized for the exploration of pan-cancer key genes and algorithm performance evaluation.
